# Epidemiological aspects of HCV infection in non-injecting drug users in the Brazilian state of Pará, eastern Amazon

**DOI:** 10.1186/1743-422X-11-38

**Published:** 2014-02-25

**Authors:** Aldemir B Oliveira-Filho, Leila Sawada, Laine C Pinto, Daiane Locks, Santana L Bahia, Jairo A A Castro, Renata B Hermes, Igor Brasil-Costa, Carlos E M Amaral, José Alexandre R Lemos

**Affiliations:** 1 Instituto de Estudos Costeiros, Campus de Bragança, Universidade Federal do Pará, Alameda Leandro Ribeiro, s/n. Aldeia, 68600-000 Bragança, Pará, Brazil; 2 Centro de Hematologia e Hemoterapia do Pará, Belém, Pará, Brazil; 3 Chiba Institute of Technology, Tsudanuma, Narashino-shi, Chiba, Japan; 4 Instituto de Ciências Biológicas, Universidade Federal do Pará, Belém, Pará, Brazil; 5 Instituto Evandro Chagas, Ananindeua, Pará, Brazil

**Keywords:** HCV, Epidemiology, Non-injecting drug, Public health, Amazon

## Abstract

**Background:**

Currently, sharing of drug paraphernalia is the main form of HCV transmission worldwide. In South America, consistent findings indicate that shared sniffing equipment is an important factor in the spread of HCV among non-injecting drug users. Epidemiological data on the status of HCV infection in illicit drug users in the Amazon region are scarce, although reports of clinical cases of hepatitis or pathologies associated with HCV infection in other population groups are numerous. Thereby, this study investigated the prevalence, genotype frequency, and epidemiological factors associated with HCV infection in non-injecting drug users in the state of Pará, eastern Amazon.

**Results:**

During 2008–2011, 300 non-injecting drug users attending drug-treatment centers participated in this study. Most non-injecting drug users were male (63.7%). The mean age was 32.5 years. The non-injecting drugs most consumed were: cannabis (15.6%), cocaine paste (21.3%), and oxi cocaine (25.7%). Tobacco (60.9%) and alcohol (79.4%) were also commonly consumed. One hundred six (35.1%; CI 95%: 29.8 - 41.1) non-injecting drug users presented anti-HCV antibodies by EIA. The HCV-RNA prevalence was 28.0% (95% CI: 20.6 - 35.8). Genotypes 1 (76.9%) and 3 (23.1%) of HCV have been identified. A multivariate analysis demonstrated that HCV infection was independently associated with the following factors: “age (≥ 35 years)”, “tattoos”, “use of a needle or syringe sterilized at home”, “shared use of drug paraphernalia”, “uses drugs for more than 5 years”, and “use of drugs everyday”.

**Conclusions:**

This study revealed a high prevalence of HCV infection in non-injecting drug users, and most infections are occasioned by genotype 1. Likely, HCV transmission is associated with the tattoos, the use of needle or syringe sterilized at home by people over the age of 35 years, and sharing, time and frequency of use of non-injecting drugs. These findings should serve as an incentive for the establishment of a program of Hepatitis C prevention and control by the local public-health authorities in order to develop effective policies and strategies for contain the spread of HCV infection.

## Background

Currently, sharing of drug paraphernalia is the main form of transmission of the hepatitis C virus (HCV) worldwide. It is estimated that more than 60% of the new cases of HCV infection recorded each year are related to the use of illicit drugs, especially by injection [1]. The prevalence of HCV infection in illicit drug users varies from 10% to 95%, reflecting the presence or absence of specific risk factors, such as the history of drug use, sharing of drug paraphernalia (needles, syringes, pipes, cans, and so on), the number of partners present during shared use, detention and the use of drugs in jail or prison, and the type of consumption: inhaled or injected [2,3]. A number of studies have shown that the sharing of drug paraphernalia is responsible for the spread of HCV among both injecting drug users and non-injecting drug users [2,4-6].

Recently in South America, consistent findings indicate that shared sniffing equipment is relevant in an important factor in the spread of HCV among non-injecting drug users [7-9]. In Brazil, epidemiological studies of HCV infection in non-injection drug user are still rare. There are few Brazilian studies investigating HCV infection in illicit drug users including both injecting and non-injecting drug users. Their HCV prevalence rates range from 5.8% to 36.2%. HCV genotype 1 predominated, while genotype 3 was the second most common in illicit-drug users in Brazil [9-14].

Epidemiological data on the status of HCV infection in illicit-drug users in the Amazon are scarce, although reports of clinical cases of hepatitis or pathologies associated with HCV infection in other population groups are numerous [15-18]. The Amazon region is the site of most of the world production of cocaine and its derivatives [19]. Commonly, the police authorities record arrests and trafficking of cocaine and its derivatives in the Brazilian portion of the Amazon region (northern Brazil). Among the states that comprise the Brazilian Amazon, the state of Pará has become known for integrating various routes of trafficking cocaine and its derivatives in Brazil and around the world. Currently, most users of illicit drugs in Pará do not use injection as the route of administration, and are infected with HCV genotype 1 [9,18]. Research has suggested that HCV infection is highly prevalent in non-injecting cocaine users, and viral transmission is likely to be associated with “shared use of paraphernalia”, “daily use of cocaine”, and a “long history of cocaine use” [9]. Given this, the aim of this study was to estimate the prevalence of HCV infection and the main genotypes circulating among non-injecting drug users in the state of Pará, and to assess the factors associated with HCV infection in these subjects.

## Results

In total, 327 illicit drug users agreed to participate in the study. However, twenty-seven patients had records of injection drug use and were excluded from analysis. According to information provided by 300 non-injecting drug users, this epidemiological study was developed. Most non-injecting drug users were male (63.7%). The mean age was 32.5 ± 10.3 years (median = 31 years). None of non-injecting drug users considered themselves to be ex-drug users (patients who reported that if they left drug-treatment centers (DTC) at the time of the interview, they would not resume using illicit drugs). Several participants (49.1%) reported having consumed more than one illicit drug during their lifetime. Thus, illicit-drug users were grouped according to the drug used most frequently. Drug preference was grouped into seven categories: cannabis (15.6%), cocaine paste (21.3%), cannabis and cocaine paste (11.7%), cocaine powder (14.0%), cocaine powder and paste (0.7%), oxi cocaine (25.7%), and crack cocaine (11.0%). Tobacco (60.9%) and alcohol (79.4%) were also commonly consumed by the subjects.

One hundred six (35.1%; CI 95%: 29.8 - 41.1) non-injecting drug users presented anti-HCV antibodies by EIA. Eighty-four (79.2%) of these 106 had HCV-RNA detected by real-time PCR. The HCV-RNA prevalence among patients was 28.0% (95% CI: 20.6 - 35.8). Furthermore, two hundred one (67.0%; CI 95%: 62.7 - 71.5) out of 300 non-injecting drug users were found by EIA to have anti-HIV antibodies. Sixty-three (31.3%; CI 95%: 26.2 - 36.2) of these 201 had anti-HCV antibodies detected by EIA, and forty-nine (24.4%; CI 95%: 18.7 - 29.9) had HCV-RNA detected by real-time PCR. The Table 1 shows more characteristics of the non-injecting drug users in this study. Using univariate analysis, several variables associated with HCV infection were identified: age (≥ 35 years), surgery, tattoos, use of a needle or syringe sterilized at home, shared use of drug paraphernalia, history of illicit drug use, and frequency of illicit drug use (daily). However, the risk factors for HCV infection became clearer only after multiple logistic regression analysis. Risk factors for HCV infection among non-injecting drug users were: age (≥ 35 years), tattoos, use of a needle or syringe sterilized at home, shared use of drug paraphernalia, history of illicit drug use (more than 5 years), and frequency of illicit drug use (daily) (Table 2). The Hosmer-Lemeshow goodness-of-fit test showed a good fit for the final model 1 (_HL_*χ*^2^ = 4.4; p = 0.6) and model 2 (_HL_*χ*^2^ = 3.1, p = 0.7).

**Table 1:** Demographic and epidemiological characteristics of non-injecting drug users attending private and public drug-treatment centers located in the Brazilian state of Pará, eastern Amazon

**Characteristics**	**Overall sample ,** ***N *** **(%)**	**Anti-HCV + *n *** **(%)**	**OR (CI 95%)**	**HCV-RNA *+ n *** **(%)**	**OR (CI 95%)**
Total	300	106 (35.3)		84 (28.0)	
Mean age (years)	32.5	36.5		37.3	
Age ≥ 35 years	114 (38.0)	63 (59.4)	4.1 (2.5 - 6.8)	44 (52.4)	2.3 (1.4 - 3.8)
Heterosexual	279 (93.0)	101 (95.3)	1.7 (0.6 - 4.9)	78 (92.9)	1.0 (0.4 - 2.6)
Anti-HIV+	201 (67.0)	63 (59.4)	1.5 (0.9 - 2.4)	49 (58.3)	0.7 (0.4 - 1.1)
Supposed route of HCV infection					
Family member or close friend infected with HCV	23 (7.7)	5 (4.7)	0.5 (0.2 - 1.3)	3 (3.5)	0.4 (0.1 - 1.2)
Recipient of blood transfusion	40 (13.3)	13 (12.5)	0.9 (0.4 - 1.8)	12 (14.3)	1.2 (0.5 - 2.3)
Surgery	139 (46.3)	58 (54.7)	1.7 (1.1 - 2.7)	44 (52.4)	1.5 (0.9 - 2.3)
Tattoos	177 (59.0)	81 (76.4)	3.3 (1.9 - 5.6)	76 (90.5)	10.9 (5.0 - 23.7)
Shared used of razor blades in the domestic environment	102 (34.0)	40 (37.7)	1.3 (0.8 - 2.1)	33 (39.3)	1.4 (0.8 - 2.3)
Shared used of blades in a barbershop/beauty salon	81 (27.0)	27 (25.5)	0.9 (0.5 - 1.5)	22 (26.2)	0.9 (0.5 - 1.7)
Use of a needle or syringe sterilized at home	72 (24.0)	33 (31.1)	1.8 (1.1 - 3.1)	28 (33.3)	2.0 (1.1 - 3.4)
Shared nail clippers	130 (43.3)	50 (47.2)	1.3 (0.8 - 2.1)	38 (42.2)	0.9 (0.5 - 1.5)
Invasive dental treatment	227 (75.7)	81 (76.4)	1.1 (0.6 - 1.9)	65 (77.4)	1.1 (0.6 - 2.1)
Shared use of drug paraphernalia	204 (68.0)	94 (88.7)	6.0 (3.1 - 11.6)	77 (91.7)	7.7 (3.4 - 17.5)
Use of drugs for more than 5 years	199 (66.3)	88 (83.0)	3.7 (2.0 - 6.5)	69 (82.1)	3.1 (1.6 - 5.7)
Use of drugs everyday	246 (82.0)	97 (91.5)	3.3 (1.5 - 6.9)	80 (95.2)	6.0 (2.1 - 17.2)
Have already been arrested in police station or prison	84 (28.0)	28 (33.3)	0.9 (0.5 - 1.4)	20 (23.8)	0.7 (0.4 - 1.3)
Use of drugs during detention	40 (13.3)	13 (32.5)	0.9 (0.4 - 1.8)	10 (11.9)	0.8 (0.4 - 1.8)
Unprotected sex	234 (78.0)	79 (74.5)	0.7 (0.4 - 1.3)	63 (75.0)	0.8 (0.4 - 1.4)
Sexual intercourse with another drug user	191 (63.7)	71 (67.0)	1.3 (0.8 - 2.1)	56 (66.7)	1.2 (0.4 -1.2)
Involvement in prostitution	157 (52.3)	50 (47.2)	0.7 (0.5 - 1.2)	36 (42.9)	0.7 (0.4 - 1.2)
More than 20 sexual partners over the past 2 years	138 (46.0)	44 (41.5)	0.8 (0.5 - 1.3)	35 (41.7)	0.8 (0.5 - 1.3)

**Table 2:** Risk factors associated with HCV infection based on EIA (model 1) and PCR (model 2) results

**Risk factors**	**Model 1**		**Model 2**	
	**OR (CI 95%)**	***p-value***	**OR (CI 95%)**	***p-value***
Age (≥ 35 years)	2.3 (1.1 - 3.9)	< 0.01	2.1 (1.2 - 4.4)	0.02
Tattoos	2.8 (1.2 - 4.1)	< 0.01	8.2 (3.9 - 17.2)	< 0.01
Use of a needle or syringe sterilized at home	1.7 (1.1 - 3.4)	0.02	2.3 (1.4 - 3.1)	< 0.01
Shared use of drug paraphernalia	5.6 (1.8 - 9.5)	< 0.01	4.1 (1.9 - 8.4)	< 0.01
Uses drugs for more than 5 years	3.3 (1.9 - 4.5)	< 0.01	3.0 (1.6 - 6.3)	< 0.01
Use of drugs everyday	2.5 (1.4 - 3.7)	< 0.01	3.8 (1.1 - 6.5)	0.01

**Figure 1:**
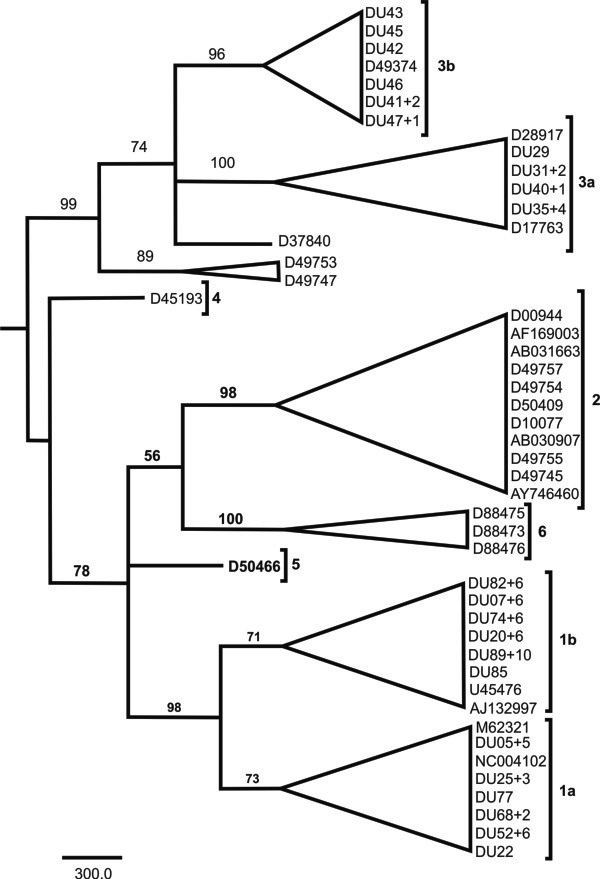
**Maximum-likelihood phylogenetic tree derived from the alignment of 341 base pairs of the 5’ UTR of the Hepatitis C virus detected in non-injecting drug users in Pará, eastern Amazon.** The topological robustness of the tree was evaluated using a bootstrap analysis with 1,000 replicates (bootstrap values of less than 50 are not shown). Drug users selected in this study can be identified by DU sample number (+ number of sequences representing).

A total of 84 nucleotide sequences of the 5’ UTR of the HCV were isolated, of which 62 (73.8%) were identical, so only one representative was maintained for the alignment. HCV genotypes were identified employing the model of Tamura-Nei adjusted by the parameters estimated through the PHYML program. The phylogenetic tree revealed a marked prevalence of genotype 1 (76.9%, 64/78), followed by genotype 3 (23.1%, 20/78). Although, the bootstrap values were not significant (< 95.0), the phylogenetic tree indicated a predominance of subtype 1b (50.0%, 42/84), followed by subtypes 1a (26.2%, 22/84), 3a (13.1%, 11/78), and 3b (10.7%, 9/78) (Figure 1).

## Discussion

In Latin America, the overall prevalence of HCV infection is about 1.23%, however it varies from country to country [20,21]. In Brazil, the prevalence of HCV infection is around 1.38%. In addition, significant differences in the prevalence of infection and distribution of HCV genotypes among Brazilian regions are reported. The northern of Brazil (Brazilian Amazon) stands out with the highest prevalence of infections and higher frequency of genotype 1 [20,22,23]. Among the risk groups for infection, illicit-drug users make up the main risk group for HCV infection in Brazil [20,22].

A consensus now exists that the use of non-injected drugs is a risk factor for the hepatitis C virus. Risk levels appear to vary substantially by location [5,6,24-26]. HCV prevalence in non-injecting drug users range from 2% to 38% in different parts of the world [3,8,11,27-29]. Epidemiological studies of illicit-drug users in the Northeast, Midwest, South and Southeast regions of Brazil (including both injecting and non-injecting drug users) have recorded prevalence of HCV infection between 5% and 36% [9-14]. The HCV prevalence recorded in the present study is within these estimated ranges, although the rate of HCV infection among non-injecting drug users in Pará was relatively high compared with the rates obtained in similar populations in different parts of the world, and extremely high and worrisome compared to the reported rates of HCV infection in other Brazilian regions. The marked prevalence of HCV-RNA in the drug-user population studied here should serve as an incentive for the establishment of a program of Hepatitis C prevention and control by the local public-health authorities. This program should include the use of non-injected drugs as a potential risk factor for transmission of HCV infection. Given the important consequence of this finding, more research on this topic is warranted in eastern Amazon.

The mechanisms of HCV transmission among non-injecting drug users are not well understood [3]. This study identified six risk factors for HCV infection. Having a tattoo was associated with HCV infection. Commonly, a tattoo is identified as a risk factor for HCV infection in illicit-drug users [2,6,8]. Likely, this reflects the lifestyle of illicit-drug users, which could include greater exposure to HCV. Furthermore, the daily use of drugs, paraphernalia sharing during drug use, and a long history of drug use were also associated with HCV infection in non-injecting drug users. Few studies have reported the presence of HCV-RNA in the nasal secretions of cocaine and crack users, indicating a possible alternative route for the transmission of the virus – the sharing of the paraphernalia used to consume these drugs [4,30]. One hypothesis to account for these cases involves intranasal transmission of HCV via contaminated implements, requiring two primary virological preconditions: the presence of blood and HCV in the nasal secretions of intranasal drug users, and the transfer of blood and HCV from the nasal cavity onto sniffing implements, which are often shared by intranasal drug users [4,30]. In Pará, HCV transmission may be associated with and powered by three risk factors: daily use of drugs, paraphernalia sharing during drug use, and a long history of drug use (more than 5 years). To prove this hypothesis, other, more-specific studies will be needed in the future.

Other risk factors for HCV infection identified in this study were age above 35 years, and use of a needle and glass syringe sterilized at home. Administering injectable medication without adequately sterilizing syringes or needles has been the main cause of HCV transmission worldwide, especially in developing countries [1,31,32]. In Brazil, disposable perforating and cutting materials for health procedures began to be used on a large scale during the second half of the 1980s. This situation, together with a lack of knowledge about HCV transmission, likely accounts for the higher prevalence of infection among Brazilians over 35, since transmission could have occurred through sharing of inadequately sterilized syringes and needles in homes with individuals who were asymptomatic and unaware that they were infected. Probably the risk factor “use of home-sterilized syringes and needles” was partly responsible for the significantly higher infection rate in the group older than 35 years. This may also indicate that longer exposure to risk factors increases the probability of infection with HCV. These two risk factors for HCV infection have also been detected in other Brazilian populations, including blood donors in the Brazilian state of Pará [12,15,17,33].

In Brazil, HCV genotype 1 predominates, followed by genotypes 3 and 2 [23,34]. In the Brazilian Amazon, HCV genotype 1 prevails in blood donors, health-care workers, illicit-drug users, patients with chronic hematological disease, and patients undergoing hemodialysis [12,15,17,18]. The results of the present study were well within this range, with a high frequency of HCV genotype 1 (1a and 1b) and the presence of genotype 3 (3a and 3b). This study also showed that in a geographic area in which the populations are predominantly infected with genotype 1, a relatively high frequency of genotype 3 can also occur [15-17,34]. This study concords with the finding of several other studies that reported the existence of a significant frequency of genotype 3 in illicit-drug users, reflecting the intrinsic characteristic of network transmission through sharing of illicit drugs and abuse of their paraphernalia [11,12,18,35,36].

The present study has limitations that should be considered in the interpretation of results. No procedures for identifying injection drug use, including those used in this study, are foolproof. The number of non-injecting drug users and their general characteristics in the state of Pará are not actually known since drug use is an illicit activity. For that reason, HCV infection was only investigated among those non-injecting drug users enrolled in private and public drug treatment centers during the study period. On the other hand, given the scarcity of epidemiological data about HCV infection among illicit-drug users in the state do Pará, our results provide background information for formulating policies and strategies for reducing risk and damage associated to the use of illicit drugs. Furthermore, the specific sexual behavior of illicit-drug users in detention was not investigated. Some studies suggest that illicit-drug use and sexual behavior during detention may be associated with HCV infection [28,37]. Finally, the 5’ UTR contains sufficient variation to resolve HCV classifications at the level of viral genotype. However, it is conserved and limited in its ability to discriminate subtypes within genotypes 1, 2, 3, 4, and 6 [38]. Thus, there is a need for sequencing of other regions of the HCV genome to improve the resolution of viral subtypes.

## Conclusions

This study revealed a high prevalence of HCV infection in non-injecting drug users. Genotype 1 has the highest frequency, and also detected the genotype 3. Likely viral transmission occurred by three mechanisms: (1) tattoos, (2) sharing needles and glass syringes at home associated with age over 35 years, and (3) a combination of shared use of paraphernalia, daily use, and a long history of non-injection drug use. These factors require special attention from public-health authorities in the Amazon region, in order to develop effective policies and strategies for contain the spread of HCV infection in the population of non-injecting drug users.

## Methods

### Sample collection

This was a cross-sectional study conducted in private and public DTC in nine municipalities in the Brazilian state of Pará (Abaetetuba, Ananindeua, Belém, Benevides, Bragança, Castanhal, Marituba, São Francisco do Pará, and Vigia), eastern Amazon. Twenty-five (78.1%) of the 32 DTC that existed during the study period collaborated with the research. These included DTC located within 350 km by road from the Centro de Hematologia e Hemoterapia do Pará (HEMOPA), Belém, PA, Brazil. Each DTC was visited at a time scheduled by the research team. The DTC had an average capacity of 15 patients. All patients who were at the center on the day when the team visited participated voluntarily. This study included participants who were using only non-injectable drugs, and excluded participants aged below 18 years. All data were collected from February 2008 to January 2011. The study was approved by the Research and Ethics Committee of the Núcleo de Medicina Tropical of the Universidade Federal do Pará, Brazil.

### Testing

Blood (5 ml) was collected in sterile tubes, allowed to clot, and centrifuged at room temperature. The resulting plasma samples were collected and stored at a temperature below 10°C. After the end of the collection period at each DTC, the samples were transported to HEMOPA. The plasma was then tested for the presence of anti-HCV antibodies by enzyme immunoassay (EIA). The Murex anti-HCV version 4.0 (Kyalami, Gauteng, South Africa) was used to measure anti-HCV antibody levels. Nonreactive samples were considered negative for HCV. Reactive sample by EIA were submitted to RNA extraction, reverse transcription, and real-time PCR with primers complementary to the conserved area of the 5’ UTR of HCV, as described elsewhere [17]. Furthermore, the HIV/HCV co-infection was evaluated. All plasma collected was tested for HIV antibody by EIA. The Murex HIV-1.2.O (Saluggia, Vercelli, Italy) was used to measure anti-HIV antibodies. All tests for diagnosis of viral infections were performed at the Laboratory of Cellular and Molecular Biology of the HEMOPA.

### Genotyping

Following the molecular screening for HCV infection using real-time PCR, samples that were positive for HCV-RNA were selected for the amplification of 5’ UTR using nested PCR [39]. The amplified fragment was sequenced in both directions using a Big Dye Cycle Sequencing standard kit and the dideoxynucleotide chain terminator method in an ABI Prism 3130 system (Applied Biosystems - Foster City, California, USA). All the nucleotide sequences obtained were edited and aligned using the BioEdit program [40]. The final alignment was entered into the DnaSP program [41] for the identification of identical nucleotide sequences. Nucleotide sequences obtained from the NCBI were added to the alignment and used for the construction of the phylogenetic tree and the differentiation of the HCV genotypes, as described elsewhere [18]. Nucleotide sequence data obtained in this study are available in GenBank under the accession numbers JN243906-JN243997.

### Questionnaire and records

The identification of the factors associated with HCV infection was based on the use of structured questionnaire (face-to-face interviews), in which the following variables were determined: gender, age, close family or friends with hepatitis C, recipient of blood transfusion, had surgery during the subject’s lifetime, had invasive dental treatment (root canal or tartar removal) during the subject’s lifetime, tattoo, shared used of razor blades in the domestic environment, shared used of blades in a barbershop, beauty salon or similar environment, use of a needle or syringe sterilized at home, shared nail clippers, types of legal drugs used during the subject’s lifetime, total time of drug use, frequency of drug use, shared use of drug paraphernalia (tubes, cans, pipes and similar), have already been arrested in police station or prison, use of drugs during detention in jails or prison, sexual orientation, unprotected sex, sexual intercourse with another drug user, involvement in prostitution, and multiple sexual partners. Furthermore, DTC structured admission records were reviewed to assess drug-use histories. The records include data on drug preference, length of drug use, sexual risk behavior, the main drugs used and their routes of administration. When clients enter treatment, each major part of the body - head, trunk, extremities and so on is examined. If needle use indications are identified, they are recorded in the client’s records. The information provided by participants through the questionnaire was compared with the records stored in DTC to identify and delete injecting drug users.

### Statistical analysis

Wilson confidence intervals were constructed for the infection prevalence estimates [42]. Simple and multiple logistic regressions were done to assess the independent effect of variables [33]. The fit of the final model was assessed using the Hosmer-Lemeshow goodness-of-fit test. Two definitions of HCV infection cases were used: (i) anti-HCV positivity shown by EIA, and (ii) HCV-RNA detection by real-time PCR. All statistical analyses were carried out using PASW Statistics version 18.0.

## Competing interests

The authors declare that they have no competing interests.

## Authors’ contributions

All authors contributed to the development of research. Study design: ABOF, JARL. Data collection and laboratory techniques: ABOF, LS, LCP, DL, SLB, JAAC, RBH, IBC, CEMA, JARL. Statistical analysis: ABOF, LS, LCP, SLB, JAAC, RBHH, IBC, CEMA. Phylogenetic analysis: ABOF, LS, DL. Writing of the manuscript: ABOF. Reviewing the manuscript: LS, LCP, SLB, DL, JAAC, RBH, IBC, CEMA, JARL. All authors read and approved the final manuscript.
